# Supplementary material for: Coral geometry and why it matters
Source: PeerJ. 2024 Feb 29;12:e17037. doi: 10.7717/peerj.17037 (PMC10909345; doi:10.7717/peerj.17037)
Supplement: Supplemental Information 1 [file peerj-12-17037-s001.pdf]

Appendix A:
Geometric relationships between coral colony parameters

Circular hemisphere

Constants
- Calcification rate, G
- Density, ρ

Radius, r_t
Linear extension rate, C_t
Surface area, $S = 2\pi r^2$
Planar area, $P = \pi r^2 = \frac{S}{2}$
Volume, $V = \frac{2}{3}\pi r^3$
Change in Mass, $\Delta M = \Delta V\rho = GS_1$

$$\Delta V = \frac{GS_1}{\rho} = V_2 - V_1 = \frac{2\pi}{3}(r_2^3 - r_1^3)$$
$$\rightarrow \quad \frac{2\pi r_2^3}{3} = \frac{2G\pi r_1^2}{\rho} - \frac{2\pi r_1^3}{3}$$
$$\rightarrow \quad r_2 = (\frac{3Gr_1^2}{\rho} + r_1^3)^{1/3}$$

Surface area productivity
$$\frac{\Delta S}{S_1} = \frac{S_2 - S_1}{S_1} = \frac{S_2}{S_1} - 1 = \frac{r_2^2}{r_1^2} - 1$$
$$\rightarrow \quad \frac{\Delta S}{S_1} = \frac{\left(\frac{3Gr_1^2}{\rho} + r_1^3\right)^{2/3}}{r_1^2} - 1$$

Planar area productivity
$$\frac{\Delta P}{S_1} = \frac{\Delta S}{2S_1} = \frac{\left(\frac{3Gr_1^2}{\rho} + r_1^3\right)^{2/3}}{2r_1^2} - \frac{1}{2}$$

Surface area to volume ratio
$$\frac{S}{V} = \frac{2\pi r^2}{\frac{2}{3}\pi r^3} = \frac{3}{r}$$

Linear extension rate
$$C_t = r_{t+1} - r_t$$
$$\rightarrow \quad r_{t+1} = C_t + r_t$$
$$G = \frac{\Delta V\rho}{S_t} = \frac{\rho(V_{t+1} - V_t)}{S_t} = \frac{\rho\left(\frac{2}{3}\pi r_{t+1}^3 - \frac{2}{3}\pi r_t^3\right)}{2\pi r_t^2} = \frac{\rho\left(r_{t+1}^3 - r_t^3\right)}{3r_t^2} = \frac{\rho\left((C_t + r_t)^3 - r_t^3\right)}{3r_1^2} = \frac{\rho(3C_t r_1^2 + 3C_t^2 r_t + C_t^3)}{3r_t^2}$$

As $r_t \rightarrow \infty$, $G \rightarrow \rho C_t$ Therefore $C_t \rightarrow \frac{G}{\rho}$

Flat disk

Constants
- Calcification rate, G
- Density, ρ
- Thickness, h

Radius, r_t
Linear extension rate, C_t
Surface area, $S = \pi r^2$
Planar area, $P = S = \pi r^2$
Volume, $V = h\pi r^2$
Change in Mass, $\Delta M = \Delta V\rho = GS_1$

$\Delta V = \frac{GS_1}{\rho} = V_2 - V_1 = h\pi(r_2^2 - r_1^2)$
$\rightarrow \quad r_2^2 = r_1^2 + \frac{Gr_1^2}{\rho h} = r_1^2(\frac{G}{\rho h} + 1)$

Surface area productivity

$$\frac{\Delta S}{S_1} = \frac{S_2 - S_1}{S_1} = \frac{S_2}{S_1} - 1 = \frac{r_2^2}{r_1^2} - 1 = \frac{r_1^2\left(\frac{G}{\rho h}+1\right)}{r_1^2} - 1 = \left(\frac{G}{\rho h} + 1\right) - 1 = \frac{G}{\rho h}$$

$\rightarrow \quad \mathbf{\frac{\Delta S}{S_1} = \frac{G}{\rho h}}$

Surface area to volume ratio

$\frac{S}{V} = \frac{\pi r^2}{h\pi r^2} = \frac{1}{h}$

Linear extension rate

$C_t = r_{t+1} - r_t$
$\rightarrow \; r_{t+1} = C_t + r_t$

$$G = \frac{\Delta V\rho}{S_t} = \frac{\rho(V_{t+1} - V_t)}{S_t} = \frac{\rho\left(h\pi r_{t+1}^2 - h\pi r_t^2\right)}{\pi r_t^2} = \frac{\rho h\left(r_{t+1}^2 - r_t^2\right)}{r_t^2} = \rho h\left(\frac{r_{t+1}^2}{r_t^2} - 1\right)$$

$\rightarrow \; r_{t+1} = r_t\sqrt{\frac{G}{\rho h} + 1}$

$\rightarrow \; C_t + r_t = r_t\sqrt{\frac{G}{\rho h} + 1}$

$\rightarrow \; C_t = r_t\left(\sqrt{\frac{G}{\rho h} + 1} - 1\right)$

As $r_t \rightarrow \infty, \quad C_t \rightarrow \infty$

<u>**Cone** (single branch)</u>

Constants

- Calcification rate, G
- Density, ρ
- Aspect ratio, α = h/r

Radius, r = h/ α
Height, h = α r (branch length)
Linear extension rate, C_t
Surface area, $S = \pi r\sqrt{h^2 + r^2} = \frac{\pi h}{\alpha}\sqrt{h^2 + \frac{h^2}{\alpha^2}} = \frac{\pi h^2}{\alpha}\sqrt{1 + \frac{1}{\alpha^2}}$ (3-sides, excluding basal attachment)
Volume, $V = \frac{1}{3}h\pi r^2 = \frac{\pi h^3}{3\alpha^2}$
Change in Mass, $\Delta M = \Delta V\rho = GS_1$

$$\Delta V = \frac{GS_1}{\rho} = V_2 - V_1 = \frac{\pi}{3\alpha^2}(h_2^3 - h_1^3)$$

$$\rightarrow \quad \frac{3G\alpha^2 S_1}{\rho\pi} = h_2^3 - h_1^3$$

$$\rightarrow \quad h_2^3 = \frac{3G\alpha^2 S_1}{\rho\pi} + h_1^3 = \frac{3Gh^2\alpha\sqrt{1+\frac{1}{\alpha^2}}}{\rho} + h_1^3$$

$$\rightarrow \quad h_2 = \sqrt[3]{\frac{3Gh^2\alpha\sqrt{1+\frac{1}{\alpha^2}}}{\rho} + h_1^3}$$

Surface area productivity

$$\frac{\Delta S}{S_1} = \frac{S_2 - S_1}{S_1} = \frac{S_2}{S_1} - 1 = \frac{\frac{\pi h_2^2}{\alpha}\sqrt{1+\frac{1}{\alpha^2}}}{\frac{\pi h_1^2}{\alpha}\sqrt{1+\frac{1}{\alpha^2}}} - 1 = \frac{h_2^2}{h_1^2} - 1 = \frac{\left(\frac{3Gh_1^2\alpha\sqrt{1+\frac{1}{\alpha^2}}}{\rho} + h_1^3\right)^{2/3}}{h_1^2} - 1 = \left(\frac{3G\alpha\sqrt{1+\frac{1}{\alpha^2}}}{\rho h_1} + 1\right)^{2/3} - 1$$

Define $L = \alpha\sqrt{1 + \frac{1}{\alpha^2}}$

$$\rightarrow \frac{\Delta S}{S_1} = \left(\frac{3GL}{\rho h_1} + 1\right)^{2/3} - 1$$

Surface area to volume ratio

$$\frac{S}{V} = \frac{\frac{\pi h^2}{\alpha}\sqrt{1+\frac{1}{\alpha^2}}}{\frac{\pi h^3}{3\alpha^2}} = \frac{3\alpha\sqrt{1+\frac{1}{\alpha^2}}}{h} = \frac{3L}{r}$$

Linear extension rate

$$C_t = h_{t+1} - h_t$$

$$G = \frac{\Delta V\rho}{S_t} = \frac{\rho(V_{t+1} - V_t)}{S_t} = \frac{\rho\left(\frac{\pi h_{t+1}^3}{3\alpha^2} - \frac{\pi h_t^3}{3\alpha^2}\right)}{\frac{\pi h_t^2}{\alpha}\sqrt{1+\frac{1}{\alpha^2}}} = \frac{\rho\left(h_{t+1}^3 - h_t^3\right)}{3h_t^2\alpha\sqrt{1+\frac{1}{\alpha^2}}} = \frac{\rho(h_{t+1}^3 - h_t^3)}{3h_t^2 L}$$

$$\rightarrow \quad \frac{3GL}{\rho}h_t^2 = h_{t+1}^3 - h_t^3$$

$$\rightarrow \quad h_{t+1} = \sqrt[3]{\frac{3GL}{\rho}h_t^2 - h_t^3}$$

$$\rightarrow \quad C_t = \sqrt[3]{\frac{3GL}{\rho}h_t^2 - h_t^3} - h_t$$

Cylinder (single branch tip)

Constants

- Calcification rate, G
- Density, ρ
- Branch radius, r

Total number of branches, β_t
Total branch length, h_t
Linear extension rate, C_t
Surface area, $S = 2\pi rh + \pi r^2$
Volume, $V = h\pi r^2$
Change in Mass, $\Delta M = \Delta V\rho = GS_1$

$$\Delta V = \frac{GS_1}{\rho} = V_2 - V_1 = h\pi r^2(h_2 - h_1)$$
$$\rightarrow \frac{GS_1}{\rho\pi r^2} = h_2 - h_1$$
$$\rightarrow h_2 = \frac{GS_1}{\rho\pi r^2} + h_1$$

Surface area productivity

$$\frac{\Delta S}{S_1} = \frac{S_2 - S_1}{S_1} = \frac{S_2}{S_1} - 1 = \frac{2\pi rh_2 + \pi r^2}{2\pi rh_1 + \pi r^2} - 1 = \frac{2h_2 + r}{2h_1 + r} - 1 = \frac{2\left(\frac{GS_1}{\rho\pi r^2} + h_1\right) + r}{2h_1 + r} - 1 = \frac{\frac{2GS_1}{\rho\pi r^2} + 2h_1 + r}{2h_1 + r} - 1$$

$$= \frac{2GS_1}{\rho\pi r^2(2h_1 + r)} = \frac{2G(2\pi rh_1 + \pi r^2)}{\rho\pi r^2(2h_1 + r)} = \frac{2G\pi r(2h_1 + r)}{\rho\pi r^2(2h_1 + r)}$$

$$\rightarrow \boldsymbol{\frac{\Delta S}{S_1} = \frac{2G}{\rho r}}$$

Surface area to volume ratio

$$\frac{S}{V} = \frac{2\pi rh + \pi r^2}{h\pi r^2} = \frac{2h + r}{hr} = \frac{2}{r} + \frac{1}{h}$$

as h→∞, $\frac{S}{V} \rightarrow \frac{2}{r}$

Linear extension rate

$$C_t = h_{t+1} - h_t$$

$$G = \frac{\Delta V\rho}{S_t} = \frac{\rho(V_{t+1} - V_t)}{S_t} = \frac{\rho(h_{t+1}\pi r^2 - h_t\pi r^2)}{2\pi rh_t + \pi r^2} = \frac{\rho r(h_{t+1} - h_t)}{2h_t + r} = \frac{\rho r(C_t + h_t - h_t)}{2h_t + r} = \frac{\rho rC_t}{2h_t + r}$$
$$\rightarrow G(2h_t + r) = \rho rC_t$$
$$\rightarrow C_t = \frac{2Gh_t}{\rho r} + \frac{G}{\rho}$$

$\rightarrow \frac{C_t}{\beta_t} = \frac{2G}{\rho r}\frac{h_t}{\beta_t} + \frac{G}{\rho\beta_t}$, where $\frac{C_t}{\beta_t}$ is average branch extension rate and $\frac{h_t}{\beta_t}$ is average branch length

As $\beta_t \rightarrow \infty$, $\frac{C_t}{\beta_t} \rightarrow \frac{2G}{\rho r}\frac{h_t}{\beta_t}$

Prolate hemispheroid (c>a)

Constants

- Calcification rate, G
- Density, ρ

- Eccentricity, $e = \sqrt{1 - \frac{a^2}{c^2}}$, c>a

  $\rightarrow \quad c = \frac{a}{\sqrt{1-e^2}}$

Equatorial radius, a=b
Polar radius, c
Surface area, $S = \pi a^2 + \frac{\pi ac}{e}\sin^{-1}e$
Volume, $V = \frac{2}{3}\pi a^2 c$
Change in Mass, $\Delta M = \Delta V\rho = GS_1$

$$\Delta V = \frac{GS_1}{\rho} = \frac{G}{\rho}\left(\pi a_1^2 + \frac{\pi a_1 c_1}{e}\sin^{-1}e\right) = \frac{G\pi}{\rho}\left(a_1^2 + \frac{a_1 c_1}{e}\sin^{-1}e\right)$$

$$V_2 = V_1 + \Delta V = \frac{2}{3}\pi a_1^2 c_1 + \frac{G\pi}{\rho}\left(a_1^2 + \frac{a_1 c_1}{e}\sin^{-1}e\right)$$

$$V_2 = \frac{2}{3}\pi a_2^2 c_2$$

$$\rightarrow \quad a_2^2 = \frac{3V_2}{2\pi c_2} = \frac{3V_2\sqrt{(1-e^2)}}{2\pi a_2}$$

$$\rightarrow \quad a_2^3 = \frac{3V_2\sqrt{(1-e^2)}}{2\pi}$$

$$\rightarrow \quad a_2 = \left(\frac{3V_2\sqrt{(1-e^2)}}{2\pi}\right)^{1/3} = \left(\left(\frac{3\sqrt{(1-e^2)}}{2\pi}\right)\left(\frac{2}{3}\pi a_1^2 c_1 + \frac{G\pi}{\rho}\left(a_1^2 + \frac{a_1 c_1}{e}\sin^{-1}e\right)\right)\right)^{1/3}$$

$$= \left(\left(\frac{3\sqrt{(1-e^2)}}{2\pi}\right)\left(\frac{2\pi a_1^3}{3\sqrt{(1-e^2)}} + \frac{G\pi}{\rho}\left(a_1^2 + \frac{a_1^2}{e\sqrt{(1-e^2)}}\sin^{-1}e\right)\right)\right)^{1/3}$$

$$= \left(\left(a_1^3 + \frac{3Ga_1^2\sqrt{(1-e^2)}}{2\rho}\left(1 + \frac{\sin^{-1}e}{e\sqrt{(1-e^2)}}\right)\right)\right)^{1/3}$$

Surface area productivity

$$\frac{\Delta S}{S_1} = \frac{S_2 - S_1}{S_1} = \frac{S_2}{S_1} - 1 = \frac{\pi a_2^2 + \frac{\pi a_2 c_2}{e}\sin^{-1}e}{\pi a_1^2 + \frac{\pi a_1 c_1}{e}\sin^{-1}e} - 1 = \frac{a_2^2 + \frac{a_2^2}{e\sqrt{(1-e^2)}}\sin^{-1}e}{a_1^2 + \frac{a_1^2}{e\sqrt{(1-e^2)}}\sin^{-1}e} - 1 = \frac{a_2^2\left(1 + \frac{\sin^{-1}e}{e\sqrt{(1-e^2)}}\right)}{a_1^2\left(1 + \frac{\sin^{-1}e}{e\sqrt{(1-e^2)}}\right)} - 1$$

$$= \frac{\left(\left(a_1^3 + \frac{a_1^2 3G\sqrt{(1-e^2)}}{2\rho}\left(1 + \frac{\sin^{-1}e}{e\sqrt{(1-e^2)}}\right)\right)\right)^{2/3}\left(1 + \frac{\sin^{-1}e}{e\sqrt{(1-e^2)}}\right)}{a_1^2\left(1 + \frac{\sin^{-1}e}{e\sqrt{(1-e^2)}}\right)} - 1 = \frac{\left(\left(a_1^3 + \frac{a_1^2 3G\sqrt{(1-e^2)}}{2\rho}\left(1 + \frac{\sin^{-1}e}{e\sqrt{(1-e^2)}}\right)\right)\right)^{2/3}}{a_1^2} - 1$$

Define $J = \left(1 + \frac{\sin^{-1}e}{e\sqrt{(1-e^2)}}\right)$

$$\rightarrow \quad \frac{\Delta S}{S_1} = \frac{\left(\left(a_1^3 + \frac{a_1^2 3GJ\sqrt{(1-e^2)}}{2\rho}\right)\right)^{2/3}}{a_1^2} - 1$$

Planar area productivity

$$\frac{\Delta P}{S_1} = \frac{P_2 - P_1}{S_1} = \frac{\pi a_2^2 - \pi a_1^2}{\pi a_1^2 + \frac{\pi a_1 c_1}{e}\sin^{-1}e} = \frac{a_2^2 - a_1^2}{a_1^2 + \frac{a_1 c_1}{e}\sin^{-1}e} = \frac{\left(\left(a_1^3 + \frac{3Ga_1^2\sqrt{(1-e^2)}}{2\rho}\left(1 + \frac{\sin^{-1}e}{e\sqrt{(1-e^2)}}\right)\right)\right)^{2/3} - a_1^2}{a_1^2 + \frac{a_1^2}{e\sqrt{(1-e^2)}}\sin^{-1}e}$$

$$= \frac{\left(\left(a_1^3 + \frac{3Ga_1^2\sqrt{(1-e^2)}}{2\rho}\left(1 + \frac{\sin^{-1}e}{e\sqrt{(1-e^2)}}\right)\right)\right)^{2/3} - a_1^2}{a_1^2\left(1 + \frac{\sin^{-1}e}{e\sqrt{(1-e^2)}}\right)}$$

$$= \frac{\left(\left(\mathbf{a_1^3} + \frac{\mathbf{a_1^2 3GJ}\sqrt{(\mathbf{1}-\mathbf{e^2})}}{\mathbf{2\rho}}\right)\right)^{\mathbf{2/3}} - \mathbf{a_1^2}}{\mathbf{a_1^2 J}}$$

Surface area to volume ratio

$$\frac{S}{V} = \frac{\pi a^2 + \frac{\pi ac}{e}\sin^{-1}e}{\frac{2}{3}\pi a^2 c} = \frac{3}{2c} + \frac{3\sin^{-1}e}{2ae}$$

Oblate hemispheroid (a>c)

Constants

- Calcification rate, G
- Density, ρ
- Eccentricity, $e = \sqrt{1 - \frac{c^2}{a^2}}$, a>c

  $\rightarrow \quad c = a\sqrt{1-e^2}$

Equatorial radius, a=b

Polar radius, c

Surface area, $S = \pi a^2 + \frac{\pi c^2}{2e}\ln\left(\frac{1+e}{1-e}\right)$

Volume, $V = \frac{2}{3}\pi a^2 c$

Change in Mass, $\Delta M = \Delta V \rho = GS_1$

$$\Delta V = \frac{GS_1}{\rho} = \frac{G}{\rho}\left(\pi a_1^2 + \frac{\pi c_1^2}{2e}\ln\left(\frac{1+e}{1-e}\right)\right) = \frac{G\pi}{\rho}\left(a_1^2 + \frac{c_1^2}{2e}\ln\left(\frac{1+e}{1-e}\right)\right)$$

$$V_2 = V_1 + \Delta V = \frac{2}{3}\pi a_1^2 c_1 + \frac{G\pi}{\rho}\left(a_1^2 + \frac{c_1^2}{2e}\ln\left(\frac{1+e}{1-e}\right)\right)$$

$$V_2 = \frac{2}{3}\pi a_2^2 c_2$$

$$\rightarrow \quad a_2^2 = \frac{3V_2}{2\pi c_2} = \frac{3V_2}{2\pi a_2\sqrt{1-e^2}}$$

$$\rightarrow \quad a_2^3 = \frac{3V_2}{2\pi\sqrt{1-e^2}}$$

$$\rightarrow \quad a_2 = \left(\frac{3V_2}{2\pi\sqrt{1-e^2}}\right)^{1/3} = \left(\frac{3\left(\frac{2}{3}\pi a_1^2 c_1 + \frac{G\pi}{\rho}\left(a_1^2 + \frac{c_1^2}{2e}\ln\left(\frac{1+e}{1-e}\right)\right)\right)}{2\pi\sqrt{1-e^2}}\right)^{1/3} = \left(\frac{\left(a_1^2 c_1 + \frac{3G}{2\rho}\left(a_1^2 + \frac{c_1^2}{2e}\ln\left(\frac{1+e}{1-e}\right)\right)\right)}{\sqrt{1-e^2}}\right)^{1/3}$$

$$= \left(\frac{\left(a_1^3\sqrt{1-e^2} + \frac{3G}{2\rho}\left(a_1^2 + \frac{a_1^2(1-e^2)}{2e}\ln\left(\frac{1+e}{1-e}\right)\right)\right)}{\sqrt{1-e^2}}\right)^{1/3} = \left(a_1^3 + \frac{3Ga_1^2}{2\rho\sqrt{1-e^2}}\left(1 + \frac{(1-e^2)}{2e}\ln\left(\frac{1+e}{1-e}\right)\right)\right)^{1/3}$$

Surface area productivity

$$\frac{\Delta S}{S_1} = \frac{S_2 - S_1}{S_1} = \frac{S_2}{S_1} - 1 = \frac{\pi a_2^2 + \frac{\pi c_2^2}{2e}\ln\left(\frac{1+e}{1-e}\right)}{\pi a_1^2 + \frac{\pi c_1^2}{2e}\ln\left(\frac{1+e}{1-e}\right)} - 1 = \frac{a_2^2 + \frac{c_2^2}{2e}\ln\left(\frac{1+e}{1-e}\right)}{a_1^2 + \frac{c_1^2}{2e}\ln\left(\frac{1+e}{1-e}\right)} - 1 = \frac{a_2^2 + \frac{a_2^2(1-e^2)}{2e}\ln\left(\frac{1+e}{1-e}\right)}{a_1^2 + \frac{a_1^2(1-e^2)}{2e}\ln\left(\frac{1+e}{1-e}\right)} - 1$$

$$= \frac{a_2^2\left(\left(1 + \frac{(1-e^2)}{2e}\right)\ln\left(\frac{1+e}{1-e}\right)\right)}{a_1^2\left(\left(1 + \frac{(1-e^2)}{2e}\right)\ln\left(\frac{1+e}{1-e}\right)\right)} - 1 = \frac{a_2^2}{a_1^2} - 1 = \frac{\left(a_1^3 + \frac{3Ga_1^2}{2\rho\sqrt{1-e^2}}\left(1 + \frac{(1-e^2)}{2e}\ln\left(\frac{1+e}{1-e}\right)\right)\right)^{2/3}}{a_1^2} - 1$$

Define $K = \frac{(1-e^2)}{2e}\ln\left(\frac{1+e}{1-e}\right)$

$$\rightarrow \quad \frac{\Delta S}{S_1} = \frac{\left(a_1^3 + \frac{3Ga_1^2(1+K)}{2\rho\sqrt{1-e^2}}\right)^{2/3}}{a_1^2} - 1 = \left(\frac{1}{a_1^2}\right)\left(a_1^3 + \frac{3Ga_1^2(1+K)}{2\rho\sqrt{1-e^2}}\right)^{2/3} - 1$$

$$\rightarrow \quad \boldsymbol{\frac{\Delta S}{S_1} = a_1^{-2}\left(a_1^3 + \frac{3Ga_1^2(1+K)}{2\rho\sqrt{1-e^2}}\right)^{2/3} - 1}$$

Planar area productivity

$$\frac{\Delta P}{S_1} = \frac{P_2 - P_1}{S_1} = \frac{\pi a_2^2 - \pi a_1^2}{\pi a_1^2 + \frac{\pi c_1^2}{2e} ln\left(\frac{1+e}{1-e}\right)} = \frac{a_2^2 - a_1^2}{a_1^2 + \frac{c_1^2}{2e} ln\left(\frac{1+e}{1-e}\right)} = \frac{\left(a_1^3 + \frac{3Ga_1^2}{2\rho\sqrt{1-e^2}}\left(1 + \frac{(1-e^2)}{2e} \ln\left(\frac{1+e}{1-e}\right)\right)\right)^{2/3} - a_1^2}{a_1^2 + \frac{a_1^2(1-e^2)}{2e} \ln\left(\frac{1+e}{1-e}\right)}$$

$$= \frac{\left(a_1^3 + \frac{3Ga_1^2}{2\rho\sqrt{1-e^2}}\left(1 + \frac{(1-e^2)}{2e} \ln\left(\frac{1+e}{1-e}\right)\right)\right)^{2/3} - a_1^2}{a_1^2\left(1 + \frac{(1-e^2)}{2e} \ln\left(\frac{1+e}{1-e}\right)\right)}$$

$$= \frac{\left(\mathbf{a_1^3 + \frac{3Ga_1^2(1+K)}{2\rho\sqrt{1-e^2}}}\right)^{\mathbf{2/3}} \mathbf{- a_1^2}}{\mathbf{a_1^2(1+K)}}$$

Surface area to volume ratio

$$\frac{S}{V} = \frac{\pi a^2 + \frac{\pi c^2}{2e} \ln\left(\frac{1+e}{1-e}\right)}{\frac{2}{3}\pi a^2 c} = \frac{3}{2c} + \frac{3c \ln\left(\frac{1+e}{1-e}\right)}{2ea^2}$$
